# On the Use of Cold as an Anæsthetic

**Published:** 1855-05

**Authors:** James Arnott


### ON THE USE OF COLD AS AN ANÆSTHETIC.

BY (1) DR. JAMES ARNOTT; and (2) others.

1. Arguing from the frequency of deaths from chloroform, Dr. James Arnott considers it imperatively necessary to substitute a safer anæsthetic, and he again urges the claims of cold to preference. In the present instance his object is to institute *a comparison between chloroform and cold as anæsthetics.* He proceeds:

"It is commonly supposed that the application of benumbing cold must be a difficult and troublesome proceeding; much more so, in both respects, than the administration of chloroform. The very contrary is the truth. Whether the cold is applied by keeping in contact with the part, for a few seconds, a refrigerating mixture of ice and salt contained in a gauze bag or a thin metallic vessel, or by touching it with a thick piece of copper that has been dipped in such a mixture, nothing can be easier; and it is impossible to fail. Different from chloroform, the anæsthetic effect is complete within a minute; and, as it has no unpleasant consequences, the surgeon is released from those protracted attentions which he is so often called upon to give in allaying the nervous symptoms that frequently follow the administration of chloroform. He requires no assistant: and, as the anæsthetic brings no new danger of its own, his mind is undisturbed during the operation, from the anxiety which he would suffer from chloroform on this account.

"The expense of either plan is so trifling, that it does not deserve mention with respect to private practice; but, with reference to hospitals, where the strictest economy is required, it may be worth while to state, that cold does not cost a twentieth part of the price of chloroform. In using a frigorific mixture for remedial purposes in dispensary practice, I have made two pennyworth of the materials answer for several cases in succession. Mr. Ferguson, of Giltspur Street, has had benumbing vessels elegantly made of silver; but, however well suited for private practice these may be, a rougher apparatus will answer. On one occasion, in employing congelation in phlebitis, I borrowed for the purpose the net which confined the hair of the attendant nurse; and the principal ingredient cost as little as the instrument which contained it, for, there being a snow-storm at the time, it was gathered from the door-step.

"The perfect safety from cold, and the anæsthesia from chloroform in the deepest operations, are the great respective advantages of these agents. Of the thousands of times intense cold has been used, not once has it been followed by any more untoward event than a slight cutaneous irritation. If the skin is merely benumbed, no redness follow the application; if congelation of the adipose matter under the skin is caused, a redness comes on, which may continue for a day or two. But, as explained elsewhere, this is the very contrary of inflammation. Instead of being a symptom of inflammation, the redness shows that a condition of the part exists, rendering inflammation impossible. And in this safety produced by congelation, there is an advantage not inferior in importance to the insensibility. For, to the erysipelas and phlebitis following surgical operations, the greater number of deaths occasioned by them is to be attributed.

"The anæsthesia from chloroform in deep operations can only be called perfect under the supposition, still contested, that the unconsciousness of the patient afterwards, that he has submitted to an operation, proceeds from having felt no pain, and not merely from having forgotten it. To judge from his struggles and cries, the latter would be the conclusion.

"The anæsthesia produced by chloroform is by no means so certain as the anæsthesia produced by cold, because, in the latter case, there is no unconsciousness. But, in deep operations, it is only the incision of the skin, which is very painful. The most eminent orthopædic practitioner of the day states, in a letter to the writer, that in the operations he is conversant with, the only source of pain is the incision of the skin; and perhaps no surgeon has had so good an opportunity of forming an opinion on this point. But all will agree, that if the sensibility of the skin were suspended, there would be very little suffering from the cutting of the deeper parts. So little, indeed, that it becomes a question whether life should be endangered by suspending it. The pain attendant on tightening the ligatures of arteries could be easily obviated by the momentary previous application of a congealing copper ball.

"Chloroform, by causing unconsciousness, prevents the patient from assisting the surgeon in his operation, and from apprising him of mistakes that may happen in its performance. The public has just been reading, with horror, accounts of attempts made to drag a stone from an unopened bladder by a foceps, introduced through the wound, and grasping both stone and bladder. But for the insensibility produced by chloroform, the screams of the unfortunate child would have at once indicated the error; and the system, perhaps, is more to be blamed than the surgeon.

"In the act of administration, and afterwards, certain inconveniencies attend both measures. Chloroform, besides producing unconsciousness, causes a sensation of choking, and is often succeeded by headach, sickness and prostration. Cold applied only to the degree of benumbing (which may often be sufficient) causes no unpleasant sensation; but when congelation is produced, there is a sense of pricking, like that caused by mustard, both at the time, and after the return of the circulation. This subsequent smarting may be entirely prevented by a moderate application of cold; and that which first takes place may be lessened, if thought worth while, by a little management.

"In recupitulating the subject, we may say, that although in deep operations, the insensibility produced by chloroform may be greater than that produced by cold (unless this were applied in the successive stages of the incision,) in all superficial operations, which constitute the immense majority, cold is superior to chloroform in the circumstances of safety, ease of application or the saving of time and trouble, certainty of producing anæsthesia, and lastly, in the power it possesses of preventing subsequent inflammation. Surely, a conscientious and humane surgeon will not allow the prejudice against novelty or innovation to outweigh so decided a superiority. Anæsthesia will no doubt, henceforth be a required element of every surgical operation, but chloroform, fortunately, is not the only mode of producing it."

2. On the other hand, cold is not always so effectual a substitute for chloroform as Dr. Arnott would have us imagine, and some cases in point are reported in the *London Practice of Medicne and Surgery.*

In one instance the reporter writes:

In several cases recently operated upon at St. Bartholmew's Hospital, trial was made as to the efficiency of congelation in preventing the pain of the incisions. Whether from a too timorous use of the means, or some other cause, the success was not so complete as could have been desired, since the patients evidently felt. Mr. Paget, however, has informed us, that in private he has, on several occasions, tried the plan, and found it to answer fully the intention of the proposer. The operations were for the removal of subcutaneous tumors, in which the main point was, that the patient should not feel the incision through the skin. In one case Mr. Paget excised a fatty tumor from the shoulder of a lady, the skin having previously been frozen; and although the incision required was four inches long, yet no pain was complained of. In proof that congelation does not hinder the subsequent healing, it may be mentioned, that in that instance a considerable part of the wound united by the first intention, and the rest of it soon closed. The mixture used was about equal parts of pounded ice and salt, enclosed in a coarse muslin bag. This was by degrees applied to the surface to be operated on, and, as the patient got used to the sensation, allowed to remain on it. The process occupied from four to six minutes, and caused no pain. Operators who make use of this plan, must recollect that the skin does not cut so crisp as natural when frozen, but like tough soap, requiring a little modification in the handling of the scalpel. The apparatus recommended by Dr. Arnott, a gauze bag, a large brass ball, a spoon, &c., is now kept by the instrument-makers; but it is very simple and may easily be extemporised without cost.

On another occasion the same reporter notices two other cases. In the first, the patient was a woman, under the care of Mr. Walton, in St. Mary's Hospital, from whom it was necessary to remove a fatty tumor from the abdominal wall. The tumor was subcutaneous, and felt quite as loose as such tumors generally are; it had the size of an adult fist, somewhat flattened. Nearly an hour was wasted in unsuccessful attempts to freeze the skin, but as this was due, of course, to mistakes in manipulation, it should not be charged against the process. At length, a mixture, properly made, was applied, and in about four minutes the requisite area of skin was frozen, as white and hard as could be wished. Without the loss of a moment's time, Mr. Walton made a deep incision through the whole required extent of skin into the tumor. This gave no pain. The tumor was seized at once, and forcible enucleation attempted. It could not, however, be extracted so easily as had been expected, and adhesions, both to the skin and to the deeper parts, required to be divided by the knife. At one part, where it appeared to have been pressed upon by the edge of the woman's stays, the adhesions between the tumor and skin were very close, and a careful division was needed. The operation lasted perhaps altogether about four minutes, and during the whole of that time, excepting the first cut in the skin, the patient was making loud cries and protestations of pain. It should be stated, that she was a remarkably quiet person, and one who did not complain for little.

In the second case, the patient was a man of middle age, under the care of Mr. Critchett in the London Hospital. The tumor was a fatty one, about the size of a large fist, and situated beneath the skin in the upper part of the front of the thigh. The freezing of the skin was very complete, nearly five minutes had been occupied in the process, and the incision into it appeared to be quite painless. The tumor had, however, rather intimate adhesions, more especially to the integuments; and the man complained much at almost every touch of the knife, excepting the first.

We had witnessed before the above, several cases of partial failure in the use of cold, but were inclined to attribute them somewhat to timorousness in its use; in these, however, it was fairly and sufficiently used. Their evidence seems clear to the effect, that, unless the tumor be so loose, and an almost instantaneous enucleation can be performed, a painless operation must not be expected. The anæsthesia does not extend at all deeper than the skin; and even in it recovery of sensibility is so rapid during the manipulations, that the division of adhesions to its surface will not

F

be painless unless made without a minute's delay. There are, doubtless, a large number of cases in which, despite these drawbacks, anæsthesia by cold may be made very useful; but the surgeon must always be careful not to promise to his patient a painless operation. As it regards the excision of tumors, it will probably, in a few instances, be completely successful, and in many others sufficiently so to afford a good pretext for avoiding the use of chloroform. It is, perhaps, adapted best of all for use in the very painful operations which it is so frequently necessary to perform on the fingers and toes. Here it can be applied from several sides at once, and a more complete and less transitory degree of anæsthesia produced.—*Ranking's Abstract—from Medical Times and Gazette.*

